# Effect of antenatal care on birth outcomes in the Gambia: A propensity score matching analysis

**DOI:** 10.1371/journal.pgph.0003880

**Published:** 2025-06-18

**Authors:** Santosh Gautam, Alasana Suso, Elizabeth Wood

**Affiliations:** 1 Keough School of Global Affairs, University of Notre Dame, Notre Dame, Indiana, United States of America; 2 Masters in Global Affairs Program, Keough School of Global Affairs, University of Notre Dame, Notre Dame, Indiana, United States of America; 3 Eck Institute for Global Health, University of Notre Dame, Notre Dame, Indiana, United States of America; University of California San Francisco, UNITED STATES OF AMERICA

## Abstract

The prevalence of low birth weight (LBW) remains disproportionately high in sub-Saharan Africa. LBW is a significant risk factor for infant mortality and is associated with long-term physical and cognitive impairments. While antenatal care (ANC) has the potential to improve birth outcomes, there is limited causal evidence on its impact in sub-Saharan settings. This study estimates the causal effects of ANC on birth weight and LBW in The Gambia using data from the 2019–20 Gambia Demographic and Health Survey (GDHS). The GDHS recorded birth weight for 8,362 children born in the five years preceding the survey; after excluding cases with missing data, the final analytical sample included 4,443 children. Multivariable regression and propensity score matching (PSM) methods were used to assess the relationship between ANC and birth outcomes, controlling for child sex and birth order, maternal age and education, household wealth, marital status, rural residence, number of children under five, and regional fixed effects. Multivariable regression estimates indicate that each additional ANC visit is associated with a 22-gram increase in birth weight and a 1.2 percentage point reduction in the likelihood of LBW. Mothers who attended four or more ANC visits (ANC 4+) had a 3.9 percentage point lower risk of delivering an LBW infant compared to those with fewer visits. PSM analysis corroborated these findings, showing that ANC 4 + was associated with a 71-gram increase in birth weight and a 4.7 percentage point reduction in LBW probability. These findings highlight the importance of health policies that promote adequate ANC coverage to reduce the high burden of LBW in resource-limited settings.

## Introduction

Newborn health, measured by birth weight, is an important marker that not only reflects an infant’s immediate well-being but is also a strong predictor of future health and development, including neonatal mortality, stunting, neurological and cognitive development, and diseases in adulthood [1]. Low birth weight (LBW), defined as infants born weighing less than 2,500 grams (5.5 pounds), according to the World Health Organization [2], remains a global public health concern with far-reaching consequences in low- and middle-income countries (LMICs). Of the 130 million infants born every year, approximately 20 million are born as LBW infants, amounting to an annual LBW prevalence of 15% globally [2]. A study in 2020 reported that the global incidence of LBW differs by region, with the highest rates observed in South Asia at 28%, followed by 13% in sub-Saharan Africa, 9% in Latin America, and 6% in East Asia and the Pacific [3]. Among sub-Saharan African (SSA) countries, Gambia, which is the setting of our study, exhibits one of the highest LBW rates, at 7.2% [4].

Intrauterine growth restriction (IUGR), which is often a comorbidity of preterm birth, can lead to premature delivery, both assisted and non-assisted, and is a common cause of LBW [5,6]. Antenatal care (ANC) plays a crucial role in the detection and management of IUGR, a condition in which a baby does not grow at the expected rate in the womb. A recent systematic review and meta-analysis of the effect of ANC on LBW in Africa found that at least one ANC visit had a statistically significant effect on birth weight [7]. This finding is consistent with recent studies showing that adequate ANC attendance is crucial for reducing LBW risks [8,9]. The WHO originally recommended at least four ANC visits during pregnancy, but this guideline was revised in 2016 to suggest at least eight ANC visits to enhance maternal and neonatal health outcomes [10]. Evidence indicates that missing these recommended ANC visits significantly increases the risk of LBW [10], a view supported by the role of ANC in delivering various therapies that improve maternal and fetal health outcomes [11]. Furthermore, LBW has been linked to missing five to eight ANC appointments, the absence of ANC in the first trimester, and a lack of essential ANC supplies [8,12]. While there is some debate over the specific impact of ANC interventions on maternal and neonatal health [13,14], research indicates that although ANC alone may not enhance birth outcomes, integrating it with other healthcare programs can significantly reduce perinatal and neonatal mortality [15,16].

Numerous studies have underscored how ANC visits are crucial for improving neonatal health outcomes in LMICs, especially for reducing the incidence of LBW. In a systematic review in 2012, regular ANC visits were significantly associated with a reduction in LBW rates, as these appointments allow timely interventions and health guidance tailored to mothers’ needs [17]. Furthermore, another systematic review highlighted that in LMICs, tailored ANC programs that include frequent and comprehensive visits can effectively decrease the rate of preterm birth [18]. These visits allow healthcare providers to manage maternal conditions such as hypertension and diabetes, which are prevalent in LMICs and linked to adverse birth outcomes. ANC visits also appear to reduce the risk of LBW by facilitating early detection and management of maternal health concerns conducive to healthy fetal development [19]. Moreover, adequate ANC attendance supports improved maternal nutrition, stress management, and cessation of harmful behaviors (e.g., tobacco or substance use), which are critical for sustaining optimal fetal weight gain [20]. Adequate ANC attendance, according to the World Health Organization (WHO), generally means attending at least four ANC visits during pregnancy. The first visit should be in the first trimester (within the first 12 weeks of gestation). Through regular assessments and education during ANC, women are better positioned to implement preventative measures, reducing the likelihood of IUGR and subsequent LBW deliveries [19]. This comprehensive care model not only addresses immediate health concerns but also sets a foundation for long-term neonatal health, underscoring the critical role of consistent and quality ANC in resource-limited settings [21].

Low birth weight in the Gambia remains a significant public health challenge. According to the 2019–2020 Gambia Demographic and Health Survey (GDHS), around 10% of infants were born as LBW infants, a small improvement from 12% reported in 2013 [22]. These findings emphasize the persistent burden of LBW in the country, with various studies highlighting the crucial role of ANC in improving birth outcomes. A prospective cohort study among women who attended an urban health center in Gambia found that regular ANC visits were linked to improved health outcomes for newborns. Women who had four or more ANC visits during pregnancy were less likely to deliver a baby with LBW than those who attended fewer visits [23]. Further evidence from Gambia underscores the positive impact of comprehensive, early ANC visits on reducing the incidence of preterm births and subsequent LBW rates within the Gambia [24].

While these studies show an association between ANC visits and birth outcomes, there is limited empirical evidence estimating the causal effects of ANC on LBW infants. Choice variables such as ANC visits are frequently influenced by maternal and household characteristics, leading to systematic differences in the characteristics of mothers who receive an adequate number of ANC visits compared to mothers with fewer than adequate ANC visits [25]. Failing to account for these differences can lead to biased estimates of ANC’s impacts on birth outcomes.

Consequently, it is essential to consider and adjust for these systematic differences when estimating the impact of treatment on outcomes. Propensity score matching (PSM) is a quasi-experimental method widely used for estimating causal effects in observational studies, particularly when randomized controlled trials are not feasible. PSM reduces the observed selection bias emanating from the systematic differences between the treated and control groups [26,27]. While existing studies highlight the association between ANC and birth weight, few employ causal inference methods to isolate the effect of ANC, particularly in low-resource settings such as The Gambia [7,22]. Our study contributes to this gap by applying the PSM method to mitigate selection bias and provide a more rigorous assessment of the impact of ANC on birth outcomes. Our objective is to estimate the causal effects of ANC on birth outcomes after adjusting for an extensive set of confounding variables. This study implements the PSM method by matching women with 4 or more ANC visits to women who did not have 4 or more ANC visits.

## Methods

### Study population and data

Gambia is one of the smallest countries in West Africa, with an estimated population of approximately 2.5 million in 2020 [28]. The infant mortality rate remains at 3.4%, and life expectancy is relatively low at 62 years. Among children under five, 5.1% experience wasting, 18% are stunted, and 12% are underweight, highlighting persistent issues of undernutrition and child health [29].

This study used individual level data from the GDHS collected in 2019–20 [22]. The GDHS is a nationally representative survey that collects information on mortality, morbidity, fertility, family planning use, and maternal & child health. The GDHS employed a stratified two-stage cluster sampling. In the first stage, 281 enumeration areas were selected with a probability proportional to their size within each sampling stratum. In the second stage, households were systematically sampled. A complete listing of households was conducted in all selected clusters, and these household lists served as the sampling frame. From each cluster, 25 households were systematically chosen, yielding a total sample of 7,025 selected households. The estimates from this sample are representative at the national level as well as for urban and rural areas and at the local government areas [22].

**Fig 1:**
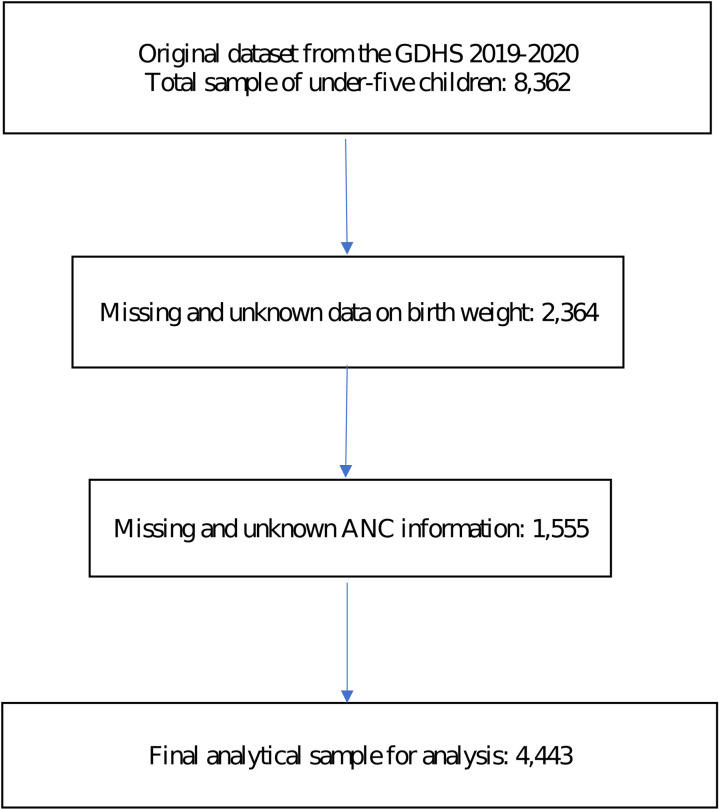
Study flow diagram.

The GDHS collected pregnancy and postnatal care information for births occurring in the year 2014 or later and has information on approximately 8,362 children. The birth weight information is available for the last two births preceding the survey. The sample of birthweight data collection is “live births in the 5 years before the survey”. This sample corresponds to liveborn babies in the 5 years before the survey, regardless of whether the baby is alive/dead at the time of the survey. Among the initial sample of 8,362 infants, birth weight data was missing or unknown for 2,364 cases (1,167 were not weighed at birth, and mothers did not know the birth weight information for 1,197 children), resulting in a sub-sample of 5,998 infants. Within this sub-sample of 5,998 infants, the ANC variable was missing for 1,555 cases, leading to a final analytical sample of 4,443 children with complete data for analysis (Fig 1). For the final sample of 4,443 infants, birth weight information was recorded for 3,844 infants from the health card, while for the remaining 599 infants, it was based on the mothers’ recall.

The ANC information was collected only for the last birth before the survey—the ANC information is available for 5,747 children. The survey questions used to collect the ANC information were as follows:

“*How many times did you receive antenatal care during this pregnancy*? “

Since the ANC variable is only available for the last birth, our analysis is restricted to the sample of the last birth in the GDHS data.

### Variables

The study has two main dependent variables: a binary indicator of low birth weight and birth weight in grams (continuous). Low birth weight is defined as a birth weight of less than 2,500 grams [29]. The main independent variables are (1) the number of ANC visits and (2) whether women completed at least four ANC visits (those with four or more ANC visits, ANC4+). Although WHO currently recommends at least eight ANC visits, The Gambia’s national guidelines and healthcare practices during the study period of the GDHS followed the previous WHO recommendation of a minimum of four visits. The GDHS collected antenatal care data for infants born in 2014 or later, who were likely to be subject to ANC 4 guidelines. Thus, we use ANC4+ as the primary threshold to reflect the policy context and healthcare practices in The Gambia. ANC4 + is a key indicator for tracking maternal health programs. The other explanatory variables were selected from the conceptual framework, a review of the relevant literature, and the availability of those variables in the GDHS. These variables included the sex and birth order of the child, maternal age and education, marital status, number of U5 children in the household, rural area, and household wealth index. The study specifically focused on singleton children under the age of five whose birth weight and sex were recorded, along with relevant information about their mothers, including age, marital status, and educational background.

### Statistical analysis

A multivariable regression model was used to examine the associations between ANC, BW, and LBW. The study used the following regression specification:


$$Y_{cmr}=\alpha_{cmr}+\beta_{1}ANC_{mr}+\beta_{2}X_{cmr}+\mu_{r}+\epsilon_{cmr}$$ (1)

where $Y_{cmr}$ is either BW in grams or a binary indicator of LBW for children *c* born to mother *m* in region *r*. ANC is defined as the number of ANC visits or a binary indicator of ANC4 + visits. We employ an ordinary least square model for both continuous outcome of birth weight (BW) and binary outcome of LBW. The linear model assumes that the LBW probability is a linear function of the regressors. We also estimated nonlinear models, such as logistic regression, allowing for nonlinear relationships between ANC and LBW. Since the results of the linear and nonlinear models are qualitatively similar, we report the results from the linear model for ease of interpretation of the coefficients. All regression models adjust for region-fixed effects to account for the time-invariant characteristics of the regions. There are eight regions in the Gambia. All analyses were conducted using non-missing data. The data were analyzed in STATA V.18 [30].

It is important to note that the estimates from equation (1) may not be the causal effects of ANC on birthweight if there is selection bias. For example, selection bias occurs when wealthier and more educated women may have more ANC visits and are also more likely to give birth to normal birth weight infants. In this case, wealthier and more educated women self-selected to have more ANC visits, thus overestimating the true effects of ANC on birth outcomes.

To address selection bias in equation (1), we employ the PSM method to estimate the causal effects of ANC on birthweight. PSM, a widely used econometric technique for establishing a causal relationship between two variables [31], offers the advantage of not depending on any specific functional form. It compares outcomes between treated and control groups within the common support. Generally, the PSM estimator for the average treatment effect (ATE) can be formulated as follows:


$$T_{ATT}=\alpha_{cmr}+\beta_{1}ANC_{mr}+\beta_{2}X_{cmr}+\mu_{r}+\epsilon_{cmr}$$

where Y(1) is the birth outcome in the ANC4 + visit group and Y(0) is the birth outcome in the < 4 ANC visit group. D is the treatment status—1 implies that women had ANC4 + visits, and 0 implies otherwise. $\tau_{ATT}$ can be interpreted as the causal impact of ANC4 + visits on birth outcomes if it satisfies the conditional independence assumption, which implies that given the vector of observed characteristics, potential outcomes are independent of treatment assignment (Y (1), Y (0)) ⊥ D|X. Therefore, it is important to specify covariates whose values do not depend on the assignment of treatment D.

Another crucial assumption is that comparisons should be made on the common support (0 < P(D = 1|X)<1), representing the overlap between the conditional probabilities. This requirement guarantees the inclusion of households whose propensity scores fall within the overlapping region of both the treatment and control groups for thorough analysis. To gauge the balance quality among the matched pairs, we examined differences in the distribution of matching covariates between the treated and control groups.

A potential concern is that missing values in the outcome variable, BW, could bias the PSM estimates, especially if the missingness is not random. If key variables like birth weight and ANC utilization are systematically missing for certain subgroups, the selected sample may not fully represent the broader population. For instance, if birth weights are disproportionately missing for home births or high-risk pregnancies, the analysis may underrepresent LBW infants, leading to an upward bias in estimated birth outcomes. To address this issue, we perform a sensitivity analysis, where we include home birth as an additional covariate in the propensity score estimation. This adjustment helps account for potential selection bias and improves the robustness of our findings.

**Fig 2:**
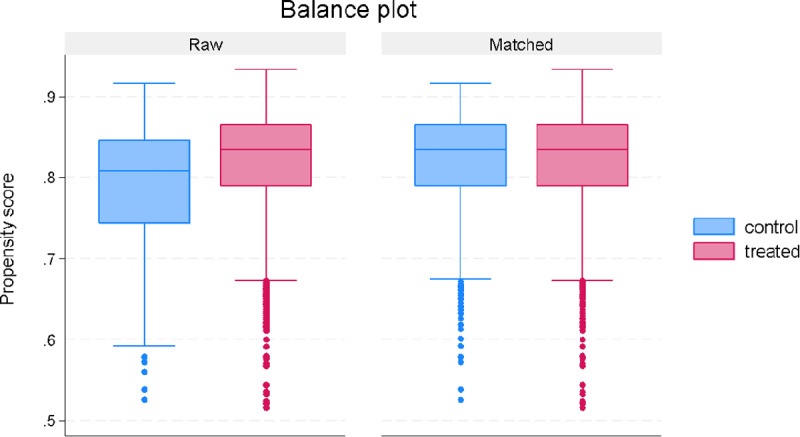
Plots of covariate balance pre- and post-matching.

**Fig 3:**
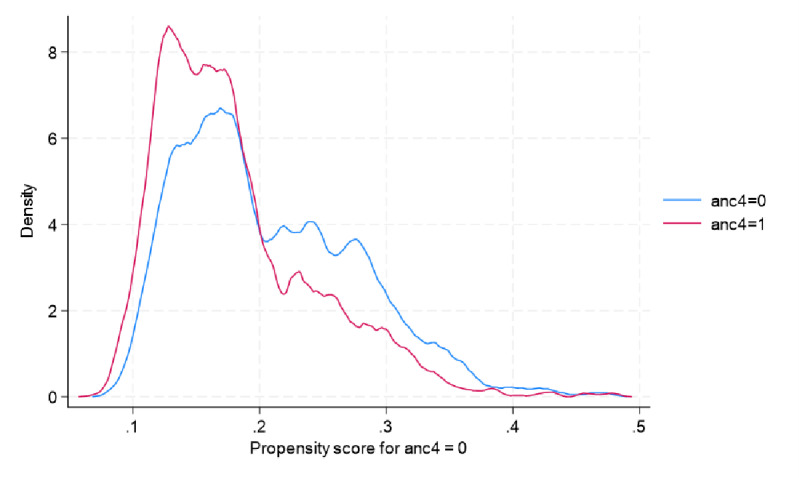
Overlap in the propensity score between the treated and control groups.

The matching quality tests are reported in Figs 2 and 3. The balance plot in Fig 2 shows that the treated and control observations are similar after matching, which is an important condition for reducing selection bias. Another crucial assumption is the overlap condition. The overlap condition implies that the density of estimated propensity scores overlaps between the treated and control observations. Fig 3 shows the overlap in the estimated density of propensity scores, indicating that the overlap assumption is satisfied.

## Results

### Descriptive analysis

Table 1 presents the summary statistics for the analytical sample used in this study. The sample size is 4,443 children. The average birth weight is 3097 grams, and approximately 8.4% of the children are low birth weight children. The mean number of ANC visits was 4.99, and the majority of the women had completed four or more ANC visits (82%). There was a statistically significant difference in the number of ANC visits between LBW and non-LBW groups. On average, mothers have completed 4.2 years of schooling; however, maternal education does not differ significantly across the two samples (p = 0.79). Approximately one-half of the sample resides in rural areas. Most women had male babies (52.46%), compared to female babies (47.54%). The wealth categories are reported in quintiles, i.e., approximately 20% of the observations belong to each of the five wealth groups ranging from poorest to richest. The wealth groups were not significantly different between the LBW and non-LBW groups.

**Table 1:** Descriptive statistics of the sample (N = 4,443).

Variable Name	Mean			Difference (*p*-value)
	All	LBW (BW < 2,500 grams)	Non-LBW (BW > 2,500 grams)	(2)-(3)
	(1)	(2)	(3)	(4)
Birth weight (grams)	3096.92	2105.82	3187.22	0.000
Low birth weight: < 2500gram (%)	8.35	–	–	–
Number of ANC visits	4.99	4.64	5.02	0.000
ANC ≥ 4 (%)	81.5	74.1	82.1	0.0007
Sex of the child (female, %)	47.5	59.2	46.4	0.000
First birth order (%)	21.2	32.1	20.2	0.000
Maternal age (years)	29.6	28.65	29.66	0.009
Maternal education	4.17	4.11	4.17	0.79
Rural (%)	50.01	47.7	50.2	0.36
Family size (< 5 years old)	3.59	3.73	3.58	0.41
Married Mothers	93.1	91.4	93.2	0.21
*Wealth Index (%)*				
Poorest	18.6	20.2	18.4	0.41
Poorer	21.7	22.3	21.6	0.75
Middle	21.2	20.2	21.3	0.62
Richer	18.9	16.2	19.2	0.13
Richest	19.6	21.02	19.4	0.47
N	4,443	371	4,072	

*Note*: *** p < 0.01, ** p < 0.05, * p < 0.1.

### Main findings

Table 2 shows the associations between ANC visits and birth outcomes estimated from linear regression model. We find that an additional ANC visit increases birth weight by 21.9 grams and reduces the probability of LBW by 1.2 percentage points. Child-level variables such as female and birth order are negatively associated with birth weight, and the regression estimates are statistically significant. Columns 3–4 further show that having more than 4 ANC visits significantly affects birth weight as well as LBW incidence. For example, if a woman had more than four ANC visits, the birth weight increased by 48 grams, and the probability of LBW decreased by 3.9 percentage points. However, these results lack a causal interpretation, and we cannot interpret these coefficients as causal effects of ANC visits on newborn birth outcomes since there are likely to be selection bias.

**Table 2:** Regression results.

	BW (grams)	LBW (BW < 2,500 grams)	BW (grams)	LBW (BW < 2,500 grams)
	(1)	(2)	(3)	(4)
ANC visits	21.89***	-0.012***		
	(1.78)	(0.002)		
ANC 4+			48.02***	-0.039***
			(9.56)	(0.008)
Female	-115.49***	0.052***	-115.92***	0.052***
	(16.99)	(0.014)	(15.87)	(0.014)
Birth order	-171.22***	0.07**	-166.04***	0.067**
	(17.84)	(0.022)	(16.77)	(0.022)
Number of U-5 children	-7.50***	0.003*	-7.97***	0.003*
	(2.11)	(0.001)	(1.92)	(0.001)
Maternal age	4.11*	0.000	4.33	0.00008
	(2.39)	(0.001)	(2.36)	(0.0005)
Maternal education	6.28**	-0.002	6.51***	-0.003
	(1.81)	(0.002)	(1.75)	(0.002)
Rural	-15.75	-0.006	-15.29	-0.005
	(22.08)	(0.013)	(23.08)	(0.013)
Marital status	-24.07	-0.010	-15.58	-0.013
	(21.98)	(0.022)	(24.78)	(0.023)
*Wealth index*				
Poorer	34.79	0.011	36.15	0.011
	(24.96)	(0.033)	(24.82)	(0.032)
Middle	18.86	-0.002	22.38	-0.004
	(13.01)	(0.008)	(13.62)	(0.007)
Richer	66.18*	-0.023*	68.27*	-0.024*
	(29.92)	(0.012)	(31.62)	(0.012)
Richest	41.34**	0.013	48.38**	0.011
	(16.20)	(0.020)	(16.46)	(0.019)
**N**	4,443	4,443	4,443	4,443

*Notes:* All models include region fixed effects. *** p < 0.01, ** p < 0.05, * p < 0.1.

To estimate the causal association between ANC visits and birth outcomes, we employed the PSM method. We use the logit model to calculate propensity scores. The covariates included in the PSM model are the sex of the child, the mother’s age, rural area, the mother’s education (years of schooling), wealth quintiles, and the number of children under five years of age in the household. Region fixed effects are included to adjust for the fixed characteristics of the regions. We used the caliper method to find the closest match for each treated observation. The caliper used for matching was 0.1. The PSM method can only be applied to binary indicators of ANC; therefore, we report the average treatment effect from the matching analysis only for the ANC4 + variable.

Table 3 shows the summary results of the covariate balance. Post-matching standardized differences are smaller for most of the variables, indicating that caliper-based matching was successful in reducing the observed differences between the treated and the control observations. A perfectly balanced covariate has a standardized difference of zero and a variance ratio of one. In Table 3, we see that our propensity score model improved the level of balance across covariates. The standardized difference has become smaller in the matched column compared to the raw column, and the variance ratios are all close to zero in column 4. For example, the variance ratio increased from 0.86 to 0.988 for the middle wealth group and from 0.621 to 0.934 for marital status, indicating improved balance in post-matching.

**Table 3:** Covariate balance summary.

	Standardized differences		Variance ratio	
	Raw (1)	Matched (2)	Raw (3)	Matched (4)
Female	-0.034	0.026	0.996	1.004
Mother’s age	0.055	-0.029	0.963	0.955
Rural	0.220	-0.030	1.030	1.003
Mothers schooling	0.065	0.065	1.119	1.098
# of under-five children	-0.026	-0.034	1.056	1.045
*Wealth index*				
Poorer	-0.058	-0.001	0.923	0.998
Middle	-0.109	-0.008	0.860	0.988
Richer	-0.052	-0.039	0.911	0.933
Richest	0.133	0.034	1.379	1.076
Birth order	0.024	0.018	1.035	1.026
Marital status	0.153	0.019	0.621	0.934
*Region*				
Kanifing	0.028	0.016	1.070	1.040
Brikama	-0.158	0.018	0.794	1.031
Mansakonko	0.072	0.022	1.250	1.065
Kerewan	0.107	-0.052	1.261	0.908
Kuntaur	0.008	-0.005	1.020	0.987
Janjanbureh	0.058	0.006	1.156	1.014
Basse	-0.070	-0.018	0.890	0.970

*Notes*: A perfectly balanced covariate will have a standardized difference close to zero and variance ratios of one.

The PSM results are shown in Table 4. Panel A shows the main results, while Panel B shows robustness checks by including home births as an additional covariate in the propensity-score model. Birth weight information was missing for infants born at home. To address potential bias from non-random sample selection, factors associated with missing birth weight, such as home births, were included as control variables in the estimation of the propensity score model in Panel B.

**Table 4:** Average treatment effects (PSM model).

	LBW (BW < 2,500 grams)	LBW (BW < 2,500 grams)	BW (grams)	BW (grams)
	ATE	ATET	ATE	ATET
	(1)	(2)	(3)	(4)
Panel A				
ANC4 +	-0.047***	-0.035**	71.18***	67.05**
	(0.015)	(0.015)	(25.81)	(27.45)
Panel B				
ANC4 +	-0.038**	-0.041**	82.93***	87.72***
	(0.016)	(0.017)	(26.04)	(27.86)
N	4,443	4,443	4,443	4,443

*Notes*: The logit model is used to estimate the propensity score. Covariates in propensity-score model: sex and birth order of the child, mother’s age, rural dummy, mother’s education, number of under-five children in the household, mother’s marital status, and region fixed effects. Panel B additionally adjusts for home births to control for missingness in birthweight.

The matching results in Panel A show that the average treatment effect (ATE) is 4.7 percentage points, indicating that women with 4 or more ANC visits had a 4.7 percentage point lower likelihood of giving birth to LBW infants. The average treatment effect on the treated (ATET) is 3.5 percentage points. The ATE and ATET effects are statistically significant. The ATE and ATET for birth weight are 71 and 67 grams, respectively. The greater magnitude of the matching results compared to the regression results demonstrates the importance of controlling for observed selection bias. The multivariable regression results underestimated the true effects of ANC on birth outcomes. Results in Panel B are qualitatively similar to the findings in Panel A. The ATE is 3.8 percentage points, while ATET is 4.1 percentage points. The effects of ANC4+ on birthweight are slightly higher than the estimates in Panel A—the ATE is 83 grams, while ATET is 88 grams. The findings in Panel B confirm that our main findings in Panel A are robust to sample selection bias due to the missingness of birth weight.

We check the sensitivity of the results by estimating inverse probability weighted regression adjustment (IPWRA) and nearest neighbor (NN) matching. NN(2) uses two neighbors to find a similar match for the treated observations. The results in Table 5, which are ATE, are qualitatively similar to the main findings in Table 4. The IPWRA model estimates a reduction in LBW incidence of 4.4 percentage points, while the NN(2) model shows an ATE size of 3.7 percentage points. Similarly, the effects on BW ranged from 62-71 grams.

**Table 5:** Sensitivity and Robustness.

	LBW (BW < 2,500 grams)	LBW (BW < 2,500 grams)	BW (grams)	BW (grams)
	IPWRA	NN(2)	IPWRA	NN(2)
	(1)	(2)	(3)	(4)
ANC4 +	-0.044***	-0.037**	71.36***	61.93***
	(0.013)	(0.015)	(22.19)	(23.56)
N	4,443	4,443	4,443	4,443

*Notes*: The logit model is used to estimate the propensity score. Covariates in the propensity-score model: sex and birth order of the child, mother’s age, rural dummy, mother’s education, number of under-five children in the household, mother’s marital status, and region fixed effects.

## Discussions

This study uses a multivariable regression model and propensity score matching method to investigate the effects of ANC utilization on birth outcomes in the Gambia. The findings underscore the significant role of ANC visits in improving birth outcomes, particularly in increasing birth weight and reducing the incidence of low birth weight. Women who had four or more ANC visits experienced an increase in the birth weight of their newborns and had a lower likelihood of giving birth to LBW babies.

Our analysis revealed a statistically significant association between the number of ANC visits and birth outcomes, with each additional ANC visit increasing the birth weight by approximately 22 grams and decreasing the probability of LBW by 1.2 percentage points. The PSM results showed that ANC utilization at 4 + visits increased birth weight by 71 grams and lowered the probability of LBW by 4.7 percentage points. Our results align with the broader literature, indicating that increased ANC utilization is associated with better neonatal health outcomes [32]. This research contributes to the discourse on maternal and newborn health outcomes, particularly BW and LBW outcomes, within the unique geographic and cultural setting of Gambia.

While the estimated increase of 22 grams in birth weight per additional ANC visit in the regression model may appear modest, it is important to consider its cumulative impact and broader implications. In a context where women are encouraged to attend multiple ANC visits, the total gain in birth weight can be more substantial—for example, attending four visits corresponds to an estimated increase of nearly 88 grams. This is particularly relevant in settings with a high burden of low birth weight, where even small increases in mean birth weight can shift the population distribution and reduce the proportion of births falling below the 2,500-gram threshold. Therefore, while modest in magnitude, the effect size observed in our study has potential clinical and public health significance, especially when scaled across the population. Additionally, the PSM estimates, which account for observed selection bias, indicate a 71-gram increase in birth weight—a gain that is both statistically and clinically meaningful in settings with a high burden of low birth weight.

The linear regression models identified a negative association between the female sex of a child and both increased BW and lower instances of LBW, confirming findings from previous studies [33,34]. An analysis from India suggested that this association might be attributed to greater maternal glucose intolerance affecting the BW of female fetuses [35]. Additionally, our multivariable regression analysis indicated a significant association between higher birth order and increased rates of LBW. This finding is consistent with a study conducted in Bangladesh that found a higher risk of LBW among individuals with higher birth orders (>3) than among those with lower birth intervals [36].

ANC use also influences facility births and the use of postnatal care services. Although this study did not examine the effects of ANC use on facility birth and postnatal service use, prior studies have demonstrated positive effects of ANC visits on facility delivery and postnatal care service utilization. Four or more ANC visits were associated with a 12% greater probability of institutional deliveries and a 10% greater probability of early postnatal check-ups in Ghana, Ethiopia, Uganda, and Kenya [37–40].

Recent discussions in maternal health research increasingly highlight the importance of quality over quantity of ANC visits, particularly in preventing LBW and other adverse pregnancy outcomes. While the number of visits is a critical factor for ensuring timely detection of risks, it is the comprehensiveness and depth of the services offered—such as nutritional screening and supplementation, infection detection and treatment, health counseling, and psychosocial support—that ultimately drive better birth outcomes [7]. Evidence indicates that interventions like iron and folic acid supplementation and ongoing education about healthy behaviors can significantly reduce LBW incidence and related perinatal complications by targeting the underlying causes of poor fetal growth [19,41]. Accordingly, international guidelines emphasize both regular attendance and the provision of high-quality, evidence-based services as indispensable pillars of effective ANC to improve pregnancy outcomes [20].

## Strengths and limitations

The primary strength of this study is to show a causal association between ANC visits and birth outcomes. To the best of our knowledge, this is one of the few studies in The Gambia to employ causal inference methods such as propensity score matching to establish causality between ANC and birth outcomes of the newborns. Furthermore, the data used in this study are nationally representative with high response rates; therefore, the findings can be generalizable throughout the Gambia and in other similar settings in sub-Saharan Africa. However, the actual sample used in the analysis is smaller than the original DHS dataset, so our findings should be interpreted with caution as a truncated sample may overestimate(underestimate) the true effects of ANC on BW(LBW). The direction of this bias depends on the characteristics of the excluded observations. If missing birth weight data is more common among women with fewer ANC visits, then the sample used in the analysis will disproportionately include mothers who received adequate ANC. This could overestimate the true positive effect of ANC on birth weight, as the truncated sample will have an underrepresentation of high-risk pregnancies with fewer ANC visits and LBW infants. Furthermore, birth weight is more frequently recorded for births in health facilities and among higher-income, more educated mothers. The final analytical sample may, therefore, overrepresent wealthier and more educated women, leading to potential confounding effects that are not fully accounted for, even after controlling for socioeconomic status. This could limit the generalizability of the study findings to more disadvantaged populations, where ANC access and birth outcomes may differ.

Among the limitations, while the PSM model accounts for selection bias based on observed characteristics, unobserved selection bias remains a concern. The PSM method can mitigate selection bias due to observables but does not alleviate selection bias caused by unobservables. Recall bias could be another factor that may bias our findings. Birth weight data were collected either from the health card or the mothers’ recall, leading to potentially inaccurate or inconsistent data. Another limitation of this study is the exclusion of observations with missing birth weight data. Birth weight information is often missing for children from lower socioeconomic groups who have a greater probability of giving birth to LBW babies. Given that preterm births and LBW infants are more likely to have missing birth weight data, our findings may underestimate the actual prevalence of LBW. This selection bias could also attenuate the estimated effects of antenatal care on LBW if the most vulnerable infants (i.e., preterm and LBW infants) were systematically excluded from the analysis.

Moreover, the exclusion of observations with missing information can underestimate the actual prevalence of LBW. The lack of data on gestational age is another potential limitation. Previous studies have shown that gestational age and preterm birth are important risk factors for LBW. Omitting these variables from the regression models is likely to bias these findings. This study is also limited by the unavailability of data in the GDHS on key maternal health indicators such as anemia status, maternal BMI, and maternal nutritional status at the time of delivery, all of which are known to influence birth outcomes. The exclusion of these variables in the empirical model may lead to residual confounding, potentially biasing the estimated associations between antenatal care and birth outcomes in our study.

A further limitation is our inability to determine the content and quality of care delivered during each ANC contact. While the number of ANC visits is an important proxy for health care utilization, it does not capture the specific components of care during the visits that are most directly linked to improved birth outcomes. The GDHS captures only the number of visits, not whether evidence‑based services—such as micronutrient supplementation, blood‑pressure monitoring, infection screening and treatment, malaria prophylaxis, or individualized counseling—were actually provided. Consequently, we cannot disentangle whether the observed effects of the ANC visits on birth outcomes are driven by contact frequency, specific service components, or the overall comprehensiveness of care. Finally, although we primarily focused on ANC4 + visits due to national guidelines, future research could explore the effects of eight or more ANC visits. However, in our sample, relatively few women had ANC8 + visits, which limited our ability to analyze this subgroup separately with sufficient statistical power.

## Conclusions

Antenatal care is a preventive form of health care that can significantly reduce complications during childbirth and can reduce maternal and neonatal mortality rates. By applying a robust causal inference framework—integrating both multivariable regression and propensity score matching—to nationally representative data, this study offers new evidence on how higher frequencies of antenatal care visits measurably improve birth outcomes in the Gambia. This methodological approach, rarely used in this context, reveals a clearer view of the protective role of ANC by mitigating confounding factors that often cloud observational research. The findings are especially valuable for guiding policy in sub-Saharan Africa, as they highlight both the quantity and quality of care as essential for meaningful gains in neonatal health. These insights underscore the urgent need for sustained investments in accessible and comprehensive ANC services. Building upon our work, future research should examine not only the frequency but also the comprehensiveness and quality of ANC services by leveraging prospective cohort designs, service‑readiness audits, and routine health‑information systems that record detailed service‑content indicators. Such data will shed light on which specific ANC components most effectively support fetal growth and neonatal survival in the Gambia and similar resource‑constrained settings, informing targeted quality‑improvement strategies.
